# Supplementary material for: Assessing species biomass contributions in microbial communities via metaproteomics
Source: Nat Commun. 2017 Nov 16;8:1558. doi: 10.1038/s41467-017-01544-x (PMC5691128; doi:10.1038/s41467-017-01544-x)
Supplement: Supplementary file 1 — Supplementary Information [file 41467_2017_1544_MOESM1_ESM.pdf]

Supplementary Table 1: Composition of the UNEVEN mock communities

Cell number and protein amount minima and maxima for bacteria marked in yellow

Label	Name and strain	Protein input per biological replicate (µg)	Protein abundance %	Cell number input per biological replicate	Cell abundance %
PD	Pseudomonas denitrificans ATCC 13867	2128.51	2.871	1930000000	0.965
BS	Bacillus subtilis 168	583.83	0.788	407100000	0.204
PaD	Paracoccus denitrificans ATCC 17741, (Beijerinck and Minkman) Davis emend. Rainey et al.	683.66	0.922	1720000000	0.860
199	Roseobacter sp. AK199	1183.43	1.596	1450000000	0.725
KF7	Pseudomonas pseudoalcaligenes KF707	863.80	1.165	603000000	0.302
CV	Chromobacterium violaceum CV026	933.08	1.259	629000000	0.315
ATN	Agrobacterium tumefaciens NTL4	4186.28	5.647	5790000000	2.895
SMS	Stenotrophomonas maltophilia SeITE02	5946.27	8.021	6630000000	3.315
Cup	Cupriavidus metallireducens CH34; (DSM 2839; LMG 1195; CIP 107179)	11504.98	15.519	26600000000	13.300
Pfl	Pseudomonas fluorescens ATCC 13525, Type strain	4964.22	6.696	3360000000	1.680
BXL	Burkholderia xenovorans LB400	321.37	0.433	2300000000	1.150
DVH	Desulfovibrio vulgaris Hildenborough	701.58	0.946	930000000	0.465
137	Staphylococcus aureus subsp. aureus Rosenbach, Smith [NCTC 10399], ATCC 13709	715.15	0.965	11900000000	5.950
259	Staphylococcus aureus subsp. aureus Rosenbach, Seattle 1945, ATCC 25923	1216.31	1.641	25300000000	12.650
Am2	Alteromonas macleodii ATCC 27126	707.47	0.954	1650000000	0.825
97 or 2197	Salmonella enterica typhimurium LT2 with Flac+ Plasmid, SA2197	4612.53	6.222	5140000000	2.570
91 or 2191	Escherichia coli K12 with Flac+ Plasmid	4290.54	5.788	6720000000	3.360
LT2	Salmonella enterica typhimurium LT2	13887.47	18.733	20800000000	10.400
H88	Salmonella enterica typhimurium LT2-H88, Rough mutant of LT2	6537.78	8.819	7500000000	3.750
HB2	Thermus Thermophilus HB27	1245.63	1.680	1610000000	0.805
CRH	Chlamydomonas reinhardtii	2962.73	3.996	225000000	0.113
M13	Phage M13	109.02	0.147	500000000	0.250
F2	Phage F2	62.04	0.084	500000000	0.250
P22	Phage P22 (HT105)	78.77	0.106	42500000000	21.250
F0	Phage F0	65.28	0.088	5850000000	2.925
ES18	Phage ES18 (H1)	65.55	0.088	725000000	0.363
841	Rhizobium leguminosarum bv. viciae 3841	680.47	0.918	975000000	0.488
VF	Rhizobium leguminosarum bv. viciae VF39	1671.03	2.254	4500000000	2.250
Ne2	Nitrosomonas europaeae ATCC 19718	60.58	0.082	90000000	0.045
Nu2	Nitrosomonas ureae Nm10	402.68	0.543	90000000	0.045
Nm2	Nitrosospira multiformis ATCC 25196	155.16	0.209	75000000	0.038
Nv	Nitrososphaera viennensis	607.41	0.819	11000000000	5.500

Supplementary Table 2: Composition of the EQUAL CELL NUMBER mock communities

Protein amount minimum and maximum for bacteria marked in yellow

Label	Name and strain	Protein input per biological replicate (µg)	Protein abundance %	Cell number input per biological replicate	Cell abundance %
PD	Pseudomonas denitrificans ATCC 13867	551.43	5.524	5.00E+08	3.571
BS	Bacillus subtilis 168	358.53	3.592	5.00E+08	3.571
PaD	Paracoccus denitrificans ATCC 17741, (Beijerinck and Minkman) Davis emend. Rainey et al.	198.74	1.991	5.00E+08	3.571
199	Roseobacter sp. AK199	408.08	4.088	5.00E+08	3.571
KF7	Pseudomonas pseudoalcaligenes KF707	716.25	7.175	5.00E+08	3.571
CV	Chromobacterium violaceum CV026	741.72	7.430	5.00E+08	3.571
ATN	Agrobacterium tumefaciens NTL4	361.51	3.622	5.00E+08	3.571
SMS	Stenotrophomonas maltophilia SeITE02	448.44	4.492	5.00E+08	3.571
Cup	Cupriavidus metalliredcens CH34; (DSM 2839; LMG 1195; CIP 107179)	216.26	2.166	5.00E+08	3.571
Pfl	Pseudomonas fluorescens ATCC 13525, Type strain	738.72	7.400	5.00E+08	3.571
BXL	Burkholderia xenovorans LB400	69.86	0.700	5.00E+08	3.571
137	Staphylococcus aureus subsp. aureus Rosenbach, Smith [NCTC 10399], ATCC 13709	30.05	0.301	5.00E+08	3.571
259	Staphylococcus aureus subsp. aureus Rosenbach, Seattle 1945, ATCC 25923	24.04	0.241	5.00E+08	3.571
Am2	Alteromonas macleodii ATCC 27126	214.38	2.148	5.00E+08	3.571
97 or 2197	Salmonella enterica typhimurium LT2 with Flac+ Plasmid, SA2197	448.69	4.495	5.00E+08	3.571
91 or 2191	Escherichia coli K12 with Flac+ Plasmid	319.24	3.198	5.00E+08	3.571
LT2	Salmonella enterica typhimurium LT2	333.83	3.344	5.00E+08	3.571
H88	Salmonella enterica typhimurium LT2-H88, Rough mutant of LT2	435.85	4.366	5.00E+08	3.571
HB2	Thermus Thermophilus HB27	386.84	3.875	5.00E+08	3.571
CRH	Chlamydomonas reinhardtii	2194.62	21.986	5.00E+08	3.571
M13	Phage M13	109.02	1.092	5.00E+08	3.571
F2	Phage F2	62.04	0.621	5.00E+08	3.571
P22	Phage P22 (HT105)	0.93	0.009	5.00E+08	3.571
F0	Phage F0	5.58	0.056	5.00E+08	3.571
ES18	Phage ES18 (H1)	45.20	0.453	5.00E+08	3.571
841	Rhizobium leguminosarum bv. viciae 3841	348.96	3.496	5.00E+08	3.571
VF	Rhizobium leguminosarum bv. viciae VF39	185.67	1.860	5.00E+08	3.571
Nv	Nitrososphaera viennensis	27.61	0.277	5.00E+08	3.571

Supplementary Table 3: Composition of the EQUAL PROTEIN AMOUNT mock communities

Cell number minimum and maximum for bacteria marked in yellow

Label	Name and strain	Protein input per biological replicate (µg)	Protein abundance %	Cell number input per biological replicate	Cell abundance %
PD	Pseudomonas denitrificans ATCC 13867	642.7	4.259	5.83E+08	0.600
BS	Bacillus subtilis 168	642.7	4.259	4.48E+08	0.462
PaD	Paracoccus denitrificans ATCC 17741, (Beijerinck and Minkman) Davis emend. Rainey et al.	642.7	4.259	1.62E+09	1.666
199	Roseobacter sp. AK199	642.7	4.259	7.87E+08	0.811
KF7	Pseudomonas pseudoalcaligenes KF707	642.7	4.259	4.49E+08	0.462
CV	Chromobacterium violaceum CV026	642.7	4.259	4.33E+08	0.446
ATN	Agrobacterium tumefaciens NTL4	642.7	4.259	8.89E+08	0.916
SMS	Stenotrophomonas maltophilia SeITE02	642.7	4.259	7.17E+08	0.738
Cup	Cupriavidus metalliredcens CH34; (DSM 2839; LMG 1195; CIP 107179)	642.7	4.259	1.49E+09	1.531
Pfl	Pseudomonas fluorescens ATCC 13525, Type strain	642.7	4.259	4.35E+08	0.448
BXL	Burkholderia xenovorans LB400	642.7	4.259	4.60E+09	4.738
137	Staphylococcus aureus subsp. aureus Rosenbach, Smith [NCTC 10399], ATCC 13709	642.7	4.259	1.07E+10	11.017
259	Staphylococcus aureus subsp. aureus Rosenbach, Seattle 1945, ATCC 25923	642.7	4.259	1.34E+10	13.771
Am2	Alteromonas macleodii ATCC 27126	642.7	4.259	1.50E+09	1.544
97 or 2197	Salmonella enterica typhimurium LT2 with Flac+ Plasmid, SA2197	642.7	4.259	7.16E+08	0.738
91 or 2191	Escherichia coli K12 with Flac+ Plasmid	642.7	4.259	1.01E+09	1.037
LT2	Salmonella enterica typhimurium LT2	642.7	4.259	9.63E+08	0.992
H88	Salmonella enterica typhimurium LT2-H88, Rough mutant of LT2	642.7	4.259	7.37E+08	0.760
HB2	Thermus Thermophilus HB27	642.7	4.259	8.31E+08	0.856
CRH	Chlamydomonas reinhardtii	642.7	4.259	4.88E+07	0.050
M13	Phage M13	62	0.411	2.84E+08	0.293
F2	Phage F2	62	0.411	5.00E+08	0.515
P22	Phage P22 (HT105)	62	0.411	3.35E+10	34.459
F0	Phage F0	62	0.411	5.56E+09	5.723
ES18	Phage ES18 (H1)	62	0.411	6.86E+08	0.706
841	Rhizobium leguminosarum bv. viciae 3841	642.7	4.259	9.21E+08	0.949
VF	Rhizobium leguminosarum bv. viciae VF39	642.7	4.259	1.73E+09	1.783
Nv	Nitrososphaera viennensis	642.7	4.259	1.16E+10	11.990

Supplementary Table 4: Statistical comparison of quantification of mock communities with metaproteomics (MP), metagenomics (MG) and 16S rRNA amplicon sequencing

	EQUAL CELL NUMBER community			EQUAL PROTEIN AMOUNT community			UNEVEN community		
**Compared to protein input**	MP	MG	16S	MP	MG	16S	MP	MG	16S
Average x fold deviation from expected value[1]	0.643	14.159	1.125	0.459	4.382	0.578	0.511	5.513	0.830
SD[2]	0.481	45.480	0.816	0.337	12.882	0.524	0.311	18.937	1.457
p-value[3]	NA	0.00000003	0.000009	NA	0.00000002	0.0002	NA	0.0000004	0.00002
**Compared to cell input**									
Average x fold deviation from expected value[1]	0.797	0.644	0.911	4.918	2.181	5.934	1.905	1.247	1.809
SD[2]	0.746	0.596	0.888	4.857	1.796	4.940	2.166	1.670	3.009
p-value[3]	NA	0.006	0.002	NA	0.0000006	0.001	NA	0.0003	0.30

[1]: The fold deviation from expected value was calculated by dividing the measured species abundances by the known relative input protein amount or cell number. This would result in a value of 1 if measurement and known input were exactly the same. For each value the deviation from this perfect accuracy value of 1 was calculated. The lowest average deviation values for each community are highlighted in green.

[2]: Standard deviation of the deviation values

[3]: T-test on the average x fold deviations for all biological replicates for each community and method. The data was log10-transformed before the test to account for the large differences in standard deviations. Comparing either metagenomics or 16S rRNA amplicon sequencing results with the metaproteomic results.

Supplementary Table 5: Overview of the metaproteomic data provided with this study

File Base Name	Run No.	Number of technical replicate runs	Number of fractions per run	Internal Experiment No.	Sample type/ species for pure cultures	LC method	Gradient length (min)
PD	3	1	1	251	*Pseudomonas denitrificans* ATCC 13867	1D	260
NV	3	1	1	251	*Nitrososphaera viennensis*	1D	260
Nm-B	3	1	1	251	*Nitrosospira multiformis* ATCC 25196	1D	260
841	3	1	1	251	*Rhizobium leguminosarum* bv. viciae 3841	1D	260
C1	1&2	2	1	253	Equal cell number mock community	1D	460
C2	1&2	2	1	253	Equal cell number mock community	1D	460
C3	1&2	2	1	253	Equal cell number mock community	1D	460
C4	1&2	2	1	253	Equal cell number mock community	1D	460
P1	1&2	2	1	253	Equal protein amount mock community	1D	460
P2	1&2	2	1	253	Equal protein amount mock community	1D	460
P3	1&2	2	1	253	Equal protein amount mock community	1D	460
P4	1&2	2	1	253	Equal protein amount mock community	1D	460
U1	1&2	2	1	253	Uneven mock community	1D	460
U2	1&2	2	1	253	Uneven mock community	1D	460
U3	1&2	2	1	253	Uneven mock community	1D	460
U4	1&2	2	1	253	Uneven mock community	1D	460
C1	3	1	12	253	Equal cell number mock community	2D	120
C2	3	1	12	253	Equal cell number mock community	2D	120
C3	3	1	12	253	Equal cell number mock community	2D	120
C4	3	1	12	253	Equal cell number mock community	2D	120
P1	3	1	12	253	Equal protein amount mock community	2D	120
P2	3	1	12	253	Equal protein amount mock community	2D	120
P3	3	1	12	253	Equal protein amount mock community	2D	120
P4	3	1	12	253	Equal protein amount mock community	2D	120
U1	3	1	12	253	Uneven mock community	2D	120
U2	3	1	12	253	Uneven mock community	2D	120
U3	3	1	12	253	Uneven mock community	2D	120
U4	3	1	12	253	Uneven mock community	2D	120
C1	4&5	2	1	253	Equal cell number mock community	1D	260
C2	4&5	2	1	253	Equal cell number mock community	1D	260
C3	4&5	2	1	253	Equal cell number mock community	1D	260
C4	4&5	2	1	253	Equal cell number mock community	1D	260
P1	4&5	2	1	253	Equal protein amount mock community	1D	260
P2	4&5	2	1	253	Equal protein amount mock community	1D	260
P3	4&5	2	1	253	Equal protein amount mock community	1D	260
P4	4&5	2	1	253	Equal protein amount mock	1D	260

File Base Name	Run No.	Number of technical replicate runs	Number of fractions per run	Internal Experiment No.	Sample type/ species for pure cultures	LC method	Gradient length (min)
					community		
U1	4&5	2	1	253	Uneven mock community	1D	260
U2	4&5	2	1	253	Uneven mock community	1D	260
U3	4&5	2	1	253	Uneven mock community	1D	260
U4	4&5	2	1	253	Uneven mock community	1D	260

Supplementary Table 6: Comparison of the detection limit and quantification accuracy for the UNEVEN mock community depending on LC method, gradient length and technical replication

Detailed data for Figure 4

		Average # of PSMs for four biological replicates normalized to %					Average # PSMs for four biological replicates					Average # identified proteins for four biological replicates				
	**Run #**	4	4&5	1	1&2	3	4	4&5	1	1&2	3	4	4&5	1	1&2	3
	**LC-run type**	1D	1D	1D	1D	2D	1D	1D	1D	1D	2D	1D	1D	1D	1D	2D
	**Gradient length (min)**	260	260	460	460	120	260	260	460	460	120	260	260	460	460	120
	**# technical replicates/fractions**	1	2	1	2	12	1	2	1	2	12	1	2	1	2	12
**Species label see Table S1**	**Protein input %**															
ATN	5.647	6.50	7.37	6.45	6.32	6.28	3590	8852	5473	10647	8293	334	379	360	420	384
Am2	0.954	0.47	0.53	0.46	0.56	0.53	257	632	392	941	693	15	18	16	22	24
BS	0.788	0.71	0.68	0.73	0.73	0.87	391	813	622	1237	1143	39	47	38	51	50
BXL	0.433	0.14	0.05	0.07	0.08	0.14	75	62	58	137	191	2	3	1	2	2
CRH	3.996	1.18	1.42	1.13	1.10	1.63	654	1704	956	1860	2148	76	85	71	87	97
CV	1.259	1.21	1.03	1.13	1.21	1.35	667	1238	962	2032	1788	50	60	54	66	71
Cup	15.519	22.24	26.09	21.17	20.77	20.49	12289	31322	17967	34985	27047	881	984	909	1022	966
DVH	0.946	0.80	0.92	0.76	0.75	0.70	440	1109	642	1256	917	36	44	37	46	41
K12	5.788	9.64	6.69	10.62	11.04	10.88	5326	8033	9018	18596	14354	177	209	195	234	234
Ne1	0.082	0.01	0.02	0.01	0.02	0.07	8	18	9	27	88	2	3	2	3	4
Nu1	0.543	0.01	0.00	0.01	0.04	0.01	6	1	9	71	20	0	1	1	1	1
NV	0.819	0.26	0.27	0.24	0.25	0.25	144	321	205	413	331	21	27	20	26	25
Nm1	0.209	0.12	0.04	0.18	0.18	0.07	64	47	156	299	98	3	3	3	3	3
PaD	0.922	0.47	0.42	0.49	0.49	0.47	262	508	418	827	615	23	29	24	31	32
ES18	0.088	0.00	0.00	0.00	0.00	0.00	0	0	0	0	0	0	0	0	0	0
F0	0.088	0.00	0.00	0.00	0.00	0.00	1	1	2	2	1	0	0	1	1	1
F2	0.084	0.00	0.00	0.00	0.00	0.00	0	0	0	0	0	0	0	0	0	0
M13	0.147	0.00	0.00	0.00	0.00	0.00	0	0	0	0	0	0	0	0	0	0
P22	0.106	0.01	0.07	0.01	0.01	0.01	8	88	5	11	13	1	1	1	1	1
PD	2.871	4.03	3.01	4.07	4.11	3.89	2225	3617	3452	6922	5133	142	171	150	186	173
Pfl	6.696	9.58	10.14	9.59	9.41	9.19	5296	12180	8143	15848	12127	381	440	403	477	431
KF7	1.165	1.91	0.97	1.90	2.13	2.37	1057	1163	1610	3592	3128	48	57	48	62	77
841	0.918	1.23	1.29	1.16	1.16	1.39	681	1554	987	1948	1837	60	74	68	80	79
VF	2.254	1.69	1.35	1.73	1.77	1.66	934	1620	1470	2978	2192	77	89	81	98	82
AK199	1.596	1.13	1.12	1.08	1.12	1.08	624	1341	918	1888	1420	69	84	71	91	85
LT2	33.773	22.43	18.60	23.09	22.98	22.55	12395	22336	19597	38713	29766	580	656	629	736	694
137	0.965	0.47	0.49	0.50	0.51	0.49	261	592	427	856	647	23	26	27	31	29
259	1.641	0.35	0.50	0.42	0.41	0.42	194	600	354	689	552	25	33	33	39	33
SMS	8.021	12.87	15.97	12.44	12.33	12.31	7111	19174	10557	20772	16244	550	623	574	670	631
HB2	1.680	0.54	0.96	0.56	0.55	0.91	298	1152	477	925	1195	45	62	55	70	91

Supplementary Table 7: Sources and cultivation conditions of microorganisms used for mock communities

Label	Name and strain	Source	Cultivation condition
PD	Pseudomonas denitrificans ATCC 13867	Dr. Jianwei Chen, University of Calgary	ATCC 3 Medium, 30° C, shaking, aerobic
BS	Bacillus subtilis 168	Dr. Jianwei Chen, University of Calgary	ATCC Medium 18, 30°C, shaking, aerobic
PaD	Paracoccus denitrificans ATCC 17741, (Beijerinck and Minkman) Davis emend. Rainey et al.	Dr. Jianwei Chen, University of Calgary	ATCC 3 Medium, 30° C, shaking, aerobic
199	Roseobacter sp. AK199	Dr. Marc Mußmann, University of Vienna	Marine Broth, 30°C, shaking, aerobic
KF7	Pseudomonas pseudoalcaligenes KF707	Dr. Sean Cameroon Booth (Turner Lab), University of Calgary	LB, 30°C, shaking, aerobic
CV	Chromobacterium violaceum CV026	Dr. Sean Cameroon Booth (Turner Lab), University of Calgary	LB, 30°C, shaking, aerobic
ATN	Agrobacterium tumefaciens NTL4	Dr. Sean Cameroon Booth (Turner Lab), University of Calgary	LB, 30°C, shaking, aerobic
SMS	Stenotrophomonas maltophilia SeITE02	Dr. Sean Cameroon Booth (Turner Lab), University of Calgary	LB, 30°C, shaking, aerobic
Cup	Cupriavidus metallireducens CH34; (DSM 2839; LMG 1195; CIP 107179)	Dr. Sean Cameroon Booth (Turner Lab), University of Calgary	LB, 30°C, shaking, aerobic
Pfl	Pseudomonas fluorescens ATCC 13525, Type strain	Dr. Sean Cameroon Booth (Turner Lab), University of Calgary	LB, 30°C, shaking, aerobic
BXL	Burkholderia xenovorans LB400	Dr. Sean Cameroon Booth (Turner Lab), University of Calgary	LB, 30°C, shaking, aerobic
DVH	Desulfovibrio vulgaris Hildenborough	Johanna Voordouw, University of Calgary	Anaerobic, 30 °C, slowly shaking, Widdel-Pfennig Medium
137	Staphylococcus aureus subsp. aureus Rosenbach, Smith [NCTC 10399], ATCC 13709	Jessica Duong (Storey Lab), University of Calgary	Tryptic Soy Broth, 37°C, shaking, aerobic
259	Staphylococcus aureus subsp. aureus Rosenbach, Seattle 1945, ATCC 25923	Jessica Duong (Storey Lab), University of Calgary	Tryptic Soy Broth, 37°C, shaking, aerobic
Am2	Alteromonas macleodii ATCC 27126	Dr. Emmo Hamann (Strous lab), University of Calgary	Marine Agar, plates, 22°C
97 or 2197	Salmonella enterica typhimurium LT2 with Flac+ Plasmid, SA2197	Dr. Kenneth Sanderson, University of Calgary	LB, 37°C, shaking, aerobic
91 or 2191	Escherichia coli K12 with Flac+ Plasmid	Dr. Kenneth Sanderson, University of Calgary	LB, 37°C, shaking, aerobic
LT2	Salmonella enterica typhimurium LT2	Dr. Kenneth Sanderson, University of Calgary	LB, 37°C, shaking, aerobic
H88	Salmonella enterica typhimurium LT2-H88, Rough mutant of LT2	Dr. Kenneth Sanderson, University of Calgary	LB, 37°C, shaking, aerobic
HB2	Thermus Thermophilus HB27	Joong-Jae Kim (Dunfield Lab), University of Calgary	Nutrient Broth, 70°C, shaking, aerobic
CRH	Chlamydomonas reinhardtii	Dr. Joenel Alcantara, University of Calgary	TAP medium, RT, with light source
M13	Phage M13	Dr. Kenneth Sanderson, University of	Host: Escherichia coli K12 with Flac+

Label	Name and strain	Source	Cultivation condition
		Calgary	plasmid
F2	Phage F2	Dr. Kenneth Sanderson, University of Calgary	Host: Escherichia coli K12 with Flac+ plasmid
P22	Phage P22 (HT105)	Dr. Kenneth Sanderson, University of Calgary	Host: Salmonella enterica typhimurium LT2
F0	Phage F0	Dr. Kenneth Sanderson, University of Calgary	Host: Salmonella enterica typhimurium LT3
ES18	Phage ES18 (H1)	Dr. Kenneth Sanderson, University of Calgary	Host: Salmonella enterica typhimurium LT4
841	Rhizobium leguminosarum bv. viciae 3841	Dr. Anupama P. Halmillawewa (Hynes Lab), University of Calgary	TY Medium, 30°C, shaking, aerobic
VF	Rhizobium leguminosarum bv. viciae VF39	Dr. Anupama P. Halmillawewa (Hynes Lab), University of Calgary	TY Medium, 30°C, shaking, aerobic
Ne2	Nitrosomonas europaeae ATCC 19718	Dr. Jessica Kozlowski (Stein Lab), University of Alberta	HEPES buffered HK medium, 22°C, in the dark, gentle shaking, aerobic
Nu2	Nitrosomonas ureae Nm10	Dr. Jessica Kozlowski (Stein Lab), University of Alberta	HEPES buffered HK medium, 22°C, in the dark, gentle shaking, aerobic
Nm2	Nitrosospira multiformis ATCC 25196	Dr. Jessica Kozlowski (Stein Lab), University of Alberta	HEPES buffered HK medium, 22°C, in the dark, gentle shaking, aerobic
Nv	Nitrososphaera viennensis	Dr. Jessica Kozlowski (Stein Lab), University of Alberta	Freshwater medium, 37°C, in the dark, not shaking, aerobic

Supplementary Table 8: Details on the mock community reference genome and protein sequences used in this study

Species and strain	Genome Accession Number(s)	Genome size (Mbp)	Protein Sequences Accession Number(s)
Pseudomonas denitrificans ATCC 13867	NC_020829.1	5.7	UP000012082
Bacillus subtilis 168	NC_000964.3	4.22	UP000001570
Paracoccus denitrificans ATCC 17741, (Beijerinck and Minkman) Davis emend. Rainey et al.	GCA_001313145.1	4.08	Used RAST to generate protein sequences. Available in the protein sequence database on PRIDE.
Roseobacter sp. AK199	Assembled from our metagenomes		Used RAST to generate protein sequences. Available in the protein sequence database on PRIDE.
Pseudomonas pseudoalcaligenes KF707	GCA_000262065.3	6.67	GCA_000262065.3
Chromobacterium violaceum CV026	Assembled from our metagenomes		Used RAST to generate protein sequences. Available in the protein sequence database on PRIDE.
Agrobacterium tumefaciens NTL4	NC_003062.2, NC_003063.2,NC_003064.2, NC_003065.3	Chr: 2.84<br>Chr: 2.08<br>Plasmid: 0.214<br>Plasmid: 0.543	UP00000813
Stenotrophomonas maltophilia SeITE02	GCF_000613205.1	4.56	GCF_000613205.1
Cupriavidus metallidurans CH34; (DSM 2839; LMG 1195; CIP 107179)	NC_007973.1, NC_007974.2, NC_007971.2, NC_007972.2	Chr: 3.93<br>Plasmid: 2.58<br>Plasmid: 0.23<br>Plasmid: 0.17	UP000002429
Pseudomonas fluorescens ATCC 13525, Type strain	IMG 2617270901	6.51	2617270901 (IMG)
Burkholderia xenovorans LB400	NC_007951.1, NC_007952.1, NC_007953.1	Chr: 4.9<br>Chr: 3.36<br>Chr: 1.47	UP000001817
Desulfovibrio vulgaris Hildenborough	NC_002937.3,NC_005863.1	Chre: 3.57<br>Plasmid: 0.202	UP000002194
Staphylococcus aureus subsp. aureus Rosenbach, Smith [NCTC 10399], ATCC 13709	ENA: SAMEA3643312	2.92	Used RAST to generate protein sequences. Available in the protein sequence database on PRIDE.
Staphylococcus aureus subsp. aureus Rosenbach, Seattle 1945, ATCC 25923	NZ_CP009361.1, NZ_CP009362.1	Chr: 2.78<br>Plasmid: 0.027	GCA_000756205.1
Alteromonas macleodii ATCC 27126	NC_018632.1	4.65	UP000006296
Salmonella enterica typhimurium LT2 with Flac+ Plasmid, SA2197	NC_003197.1, NC_003277.1, F Plasmid: AP001918.1	Chr: 4.86 Plasmid: 0.094	UP000001014
Escherichia coli K12 with Flac+ Plasmid	NC_000913.3, AP001918.1	Chr: 4.64	UP000000625
Salmonella enterica	NC_003197.1,	Chr: 4.86	UP000001014

**Species and strain**	**Genome Accession Number(s)**	**Genome size (Mbp)**	**Protein Sequences Accession Number(s)**
typhimurium LT2	NC_003277.1	Plasmid: 0.094	
Salmonella enterica typhimurium LT2-H88, Rough mutant of LT2	NC_003197.1, NC_003277.1	Same as LT2	Same as LT2
Thermus Thermophilus HB27	NC_005835.1, NC_005838.1	Chr: 1.89<br>Plasmid: 0.23	UP000000592
Chlamydomonas reinhardtii	GCF_000002595.1	Mito: 0.016<br>Chloropl: 0.2<br>Nucl: 120.19	GCF_000002595.1
Phage M13	NC_003287.2	0.00641	UP000002111
Phage F2	NC_001417.2, F2 Protein Coat: M24832.1	0.00357	UP000002127
Phage P22 (HT105)	NC_002371.2	0.04172	UP000007960
Phage F0	NC_005282.1	0.08616	UP000009070
Phage ES18 (H1)	NC_006949.1	0.0469	UP000000970
Rhizobium leguminosarum bv. viciae 3841	NC_008380.1, NC_008382.1, NC_008383.1, NC_008379.1, NC_008381.1, NC_008384.1,NC_008378.1	Chr: 5.06<br>Plasmid: 0.15<br>Plasmid: 0.15<br>Plasmid: 0.35<br>Plasmid: 0.49<br>Plasmid: 0.68<br>Plasmid 0.87	UP000006575
Rhizobium leguminosarum bv. viciae VF39	NZ_ATYQ00000000.1	7.58	GCA_000427765.1
Nitrosomonas europaeae ATCC 19718	NC_004757.1	2.81	UP000001416
Nitrosomonas ureae Nm10	NZ_CP013341.1	3.31	UP000056699
Nitrosospira multiformis ATCC 25196	NC_007614.1, NC_007615.1, NC_007616.1, NC_007617.1	Chr: 3.18<br>Plasmid: 0.02<br>Plasmid: 0.02<br>Plasmid: 0.01	UP000002718
Nitrososphaera viennensis	CP007536.1	2.53	GCA_000698785.1
