# Supplementary material for: Assessing species biomass contributions in microbial communities via metaproteomics
Source: Nat Commun. 2017 Nov 16;8:1558. doi: 10.1038/s41467-017-01544-x (PMC5691128; doi:10.1038/s41467-017-01544-x)
Supplement: Supplementary file 4 — Description of Additional Supplementary Information [file 41467_2017_1544_MOESM4_ESM.pdf]

File Name: Supplementary Data 1

Description: Comparison of different proteomic quantification methods using mock community samples
